# Identifying subtype-specific associations between gene expression and DNA methylation profiles in breast cancer

**DOI:** 10.1186/s12920-017-0268-z

**Published:** 2017-05-24

**Authors:** Garam Lee, Lisa Bang, So Yeon Kim, Dokyoon Kim, Kyung-Ah Sohn

**Affiliations:** 1 Department of Software and Computer Engineering, Ajou University, Suwon, 16499 South Korea; 2 Biomedical & Translational Informatics Institute, Geisinger Health System, Danville, PA USA; 3 The Huck Institute of the Life Sciences, Pennsylvania State University, University Park, PA USA

## Abstract

**Background:**

Breast cancer is a complex disease in which different genomic patterns exists depending on different subtypes. Recent researches present that multiple subtypes of breast cancer occur at different rates, and play a crucial role in planning treatment. To better understand underlying biological mechanisms on breast cancer subtypes, investigating the specific gene regulatory system via different subtypes is desirable.

**Methods:**

Gene expression, as an intermediate phenotype, is estimated based on methylation profiles to identify the impact of epigenomic features on transcriptomic changes in breast cancer. We propose a kernel weighted l1-regularized regression model to incorporate tumor subtype information and further reveal gene regulations affected by different breast cancer subtypes. For the proper control of subtype-specific estimation, samples from different breast cancer subtype are learned at different rate based on target estimates. Kolmogorov Smirnov test is conducted to determine learning rate of each sample from different subtype.

**Results:**

It is observed that genes that might be sensitive to breast cancer subtype show prediction improvement when estimated using our proposed method. Comparing to a standard method, overall performance is also enhanced by incorporating tumor subtypes. In addition, we identified subtype-specific network structures based on the associations between gene expression and DNA methylation.

**Conclusions:**

In this study, kernel weighted lasso model is proposed for identifying subtype-specific associations between gene expressions and DNA methylation profiles. Identification of subtype-specific gene expression associated with epigenomic changes might be helpful for better planning treatment and developing new therapies.

## Background

Altered gene expression that regulates cell growth and differentiation is a major component to transform normal cell into a cancer cell [1]. Expression of tumor suppressor genes or oncogenes affects many proteins that are turned on or off via gene silencing or gene activation, further inhibiting cell division and development and promoting the malignant phenotype of cancer cells, respectively [1]. In addition, other types of genomic data, including somatic mutations, copy number alterations (CNA), DNA methylation, or miRNA expression, are associated with cancer [2–5]. However, there are still huge gaps between genomic/epigenomic data and cancer as a phenotypic end-point to fully understand the complex mechanisms of cancer. Thus, transcriptomic changes could serve as a proxy to capture phenotypic variations in human cancer as an intermediate phenotype [6–8]. To identify genomics changes that are associated with functional changes in cancer, there have been many integrative analyses between genomic data and transcriptomic data. Many expression quantitative trait loci (eQTL) studies in cancer have been conducted to identify genomic variations that could explain the variance of the expression traits [9, 10]. In addition, associations between CNA data as a structural change and gene expression data were investigated to search genes associated with gene dosage in cancer [11, 12].

DNA methylation is one of the major mechanisms of epigenetic regulation that promotes or inhibits cancer related genes [13]. Cytosine methylation of CpG islands, which is the most common type of DNA methylation, occurs genome-wide in protein coding regions, including promoters, 5’ and 3’-UTRs, or exons, as well as in the intergenic regions [13]. CpG methylations are likely to occur in promoter regions located close to the start of transcription, and hypermethylation in the promoter regions is negatively associated with the transcript level [14]. For example, the hypermethylation of tumor suppressor genes, which is associated with their inhibition of transcription, is recognized as one of the key features of cancer pathogenesis [13]. On the contrary, CpG methylations in gene body regions are likely to be positively associated with transcript level [14]. To search relationships between epigenetic changes and transcriptomic changes in cancer, many integrative studies have been reported [15–18]. Recently, numerous prediction models using machine learning to estimate the consequence of epigenetic changes on gene expression have been developed [19–21]. In the previous study from Karlic *et al* [20], it reveals that predicting gene expression levels based on histone modifications is applicable. In addition, Cheng *et al* [21] has improved overall prediction performance of estimating gene expression levels. However, cancer is an extremely heterogeneous disease. Each cancer has many distinct subtypes and there are different genomic patterns based on different subtypes in cancer. Thus, there is a need to investigate subtype-specific epigenetic regulation mechanism in cancer.

**Fig. 1:**
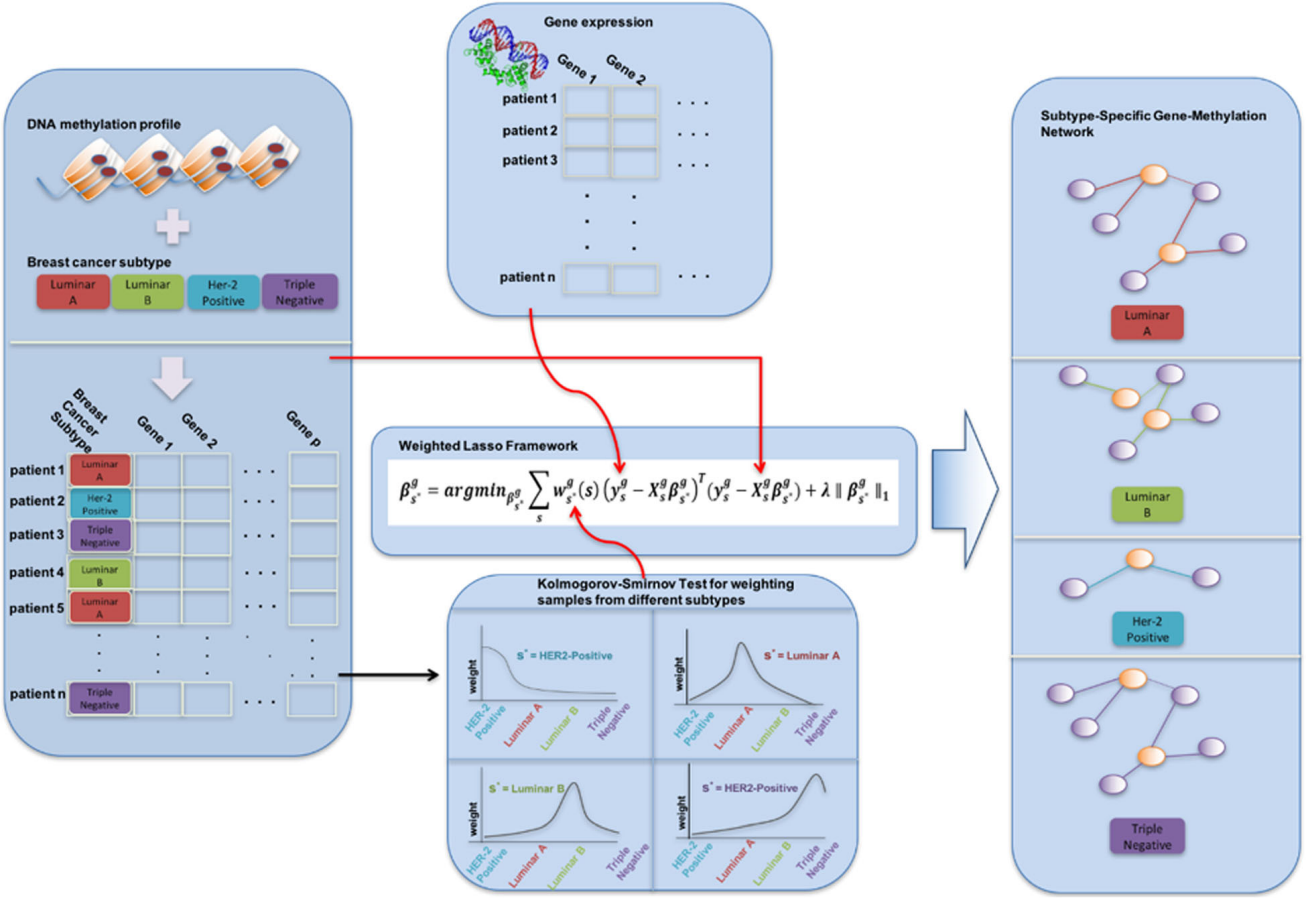
Overview of the proposed framework for identifying subtype-specific association patterns. For target gene estimation, our weighted lasso framework requires a covariate matrix and subtype information on samples. Note that four colors in Breast Cancer Subtype field correspond to subtypes, Luminal A, Luminal B, *HER2* positive, and Triple Negative, respectively. With two inputs mixed from a kernel method, target genes in each of the subtypes are estimated based on DNA methylation features

In this study, we propose a novel method that incorporates subtype information to better explain gene expression variability based on methylation profiles. Inference of subtype-specific association patterns between gene expressions and DNA methylation features is challenging because the number of available samples in each subtype may not be large enough to produce reliable estimations. Therefore, separate estimation of association patterns on each subtype is not typically feasible. We address this issue by employing a kernel weighted lasso model that can incorporate information from samples in different subtypes while allowing subtype-specific estimations. As illustrated in Fig. 1, our proposed method requires two types of input: covariate matrix as commonly used in linear regression, and prior knowledge for differentiating observations. For the proper use of prior knowledge, a weighted kernel method is applied to be mixed with independent variables. Finally, the weighted lasso framework provides subtype-specific estimation method for gene expression level. To test the utility of the proposed method, we applied it to a breast cancer data set from The Cancer Genome Atlas (TCGA). TCGA has provided unprecedented opportunities to better understand the genetic architecture of cancers through integrating multi-omics data [7, 22–30]. In particular, breast cancer has well-known distinct subtypes, including luminal A, luminal B, *HER2* positive, and triple negative or basal-like type. Depending on subtypes in breast cancer, treatment and therapy approaches are different. Thus, identification of subtype-specific gene expressions associated with epigenetic changes might be useful for better planning treatment and developing new therapies.

## Methods

### Dataset

DNA methylation and gene expression data of 437 patients in breast cancer were obtained from TCGA (https://gdc.nci.nih.gov/). Gene expression data from RNA-seq consisted of 20,502 unique genes with upper quartile normalized RNA-Seq by Expectation-Maximization (RSEM) count estimates [31]. DNA methylation data was retrieved as a gene-level feature by choosing the probe least correlated with gene expression when genes were mapped with multiple methylation probes, from 485,577 methylation probes to 19,943 [25]. Numerical data were normalized by log (T + 1) where T was DNA methylation or gene expression level. Since the size of features and target genes to estimate was too large, part of them were filtered out. First, genes that were not members in any KEGG pathways were removed. This implies that we used genes that are known to be involved in certain molecular processes. Second, we removed trivial genes for which more than half of patient records were zero due to the unrecorded elements or experimental failures to measure expression level. Finally, we had 4,237 DNA methylation genes as features, and 4,062 genes for target gene estimation. Along with numerical data, breast cancer subtype information of all patients was also provided. The 437 observations are divided into four subtypes as shown in Table 1.

**Table 1:** Number of samples per subtype

HER2 Positive	Luminal A	Luminal B	Triple Negative	Total
16	306	42	73	437

### Background on *L*_1_-regularized linear regression

Suppose we have data (***x*** ^***i***^, ***y***_***i***_) for ***i*** = 1, 2, …, ***n***, where ***x*** ^***i***^ = (***x***_***i***1_ , …, ***x***_***ip***_)^***T***^ ∈ ℝ^***p***^ is a feature vector and ***y***_***i***_ ∈ ℝ is response for the ***i*** -th observation. In a linear regression model to predict the response based on the features, the ordinary least squares (OLS) estimates for the regression coefficients ***β*** ∈ ℝ^***p***^ are obtained by minimizing residual squared error as follows.

$$ \boldsymbol{\beta} =\boldsymbol{argmi}{\boldsymbol{n}}_{\boldsymbol{\beta}}\ {\left(\boldsymbol{y}-\boldsymbol{X}\boldsymbol{\beta } \right)}^{\boldsymbol{T}}\left(\boldsymbol{y}-\boldsymbol{X}\boldsymbol{\beta } \right) $$

where ***X*** ∈ ℝ^***n*** × ***p***^ is the covariate matrix for features, and ***y*** = (***y***_1_, …, ***y***_***n***_)^***T***^. However, OLS estimates often have low bias but large variance; prediction accuracy can sometimes be improved by setting to 0 some coefficients [32]. Also, among a large number of predictors, determining a smaller subset of features that exhibits the strongest effects is more desirable. To satisfy the requirement, *L*_*1*_-regularized linear regression model, which is called lasso was proposed [32]. The lasso estimates are defined as:

$$ \boldsymbol{\beta} =\boldsymbol{argmi}{\boldsymbol{n}}_{\boldsymbol{\beta}}{\left(\boldsymbol{y}-\boldsymbol{X}\boldsymbol{\beta } \right)}^{\boldsymbol{T}}\left(\boldsymbol{y}-\boldsymbol{X}\boldsymbol{\beta } \right)+\boldsymbol{\lambda} \parallel \boldsymbol{\beta} {\parallel}_1 $$

where ***λ*** is a parameter for regulating the number of non-zero entries in the estimated ***β***, and hence the sparsity of the coefficients. The parameter ***λ*** is typically determined through cross-validation. For the selection of a small number of effective features, ***L***_1_ -regulurized linear regression is known to be efficient.

### Kernel weighted lasso for subtype-specific association network estimation

Gene expression, as an intermediate phenotype, is estimated based on DNA methylation profiles to identify the impact of epigenome on transcriptome in breast cancer. For understanding genomic mechanisms resulted from various breast cancer subtypes, we use weighted lasso with some modification in which subtype information of patients is incorporated using kernel-based weighting method. We concentrate on utilizing samples from various types of data. Especially in terms of small sample size problem, which is frequently encountered in the field of computational biology, our proposed method is exploited to enlarge the sample size by employing different types of samples. For example, samples resulted from a variety of breast cancer subtypes such as Luminal A, Luminal B, and Triple negative can be used in estimating a target gene whose subtype is HER2 positive.

As a response vector, ${\boldsymbol{y}}^{\boldsymbol{g}}\in {\mathrm{\mathbb{R}}}^{{\boldsymbol{n}}_{\boldsymbol{s}}}$ denotes gene expression level of target gene ***g***, where ***n***_***s***_ is the number of samples whose subtype is ***s***. The covariate matrix ${\boldsymbol{X}}_{\boldsymbol{s}}^{\boldsymbol{g}}\in {\mathrm{\mathbb{R}}}^{{\boldsymbol{n}}_{\boldsymbol{s}}\times {\boldsymbol{p}}^{\boldsymbol{g}}}$ is DNA methylation profile from samples whose subtype is ***s***, where ***p*** ^***g***^ is the number of features for estimating target gene ***g***. Note that the feature matrix ***X***_***s***_^***g***^ is changed over target genes, because for each target gene, we select DNA methylation features that are more likely to affect the target gene based on prior knowledge. Specifically, only DNA methylation genes that are extracted from those KEGG pathways where the target gene belongs to are selected for estimation. Finally, with modified lasso model, our proposed method for estimating the coefficients ${\boldsymbol{\beta}}_{{\boldsymbol{s}}^{*}}^{\boldsymbol{g}}$ for subtype ***s*** ^*^ is defined as:

$$ {\boldsymbol{\beta}}_{{\boldsymbol{s}}^{*}}^{\boldsymbol{g}}=\boldsymbol{argmi}{\boldsymbol{n}}_{{\boldsymbol{\beta}}_{{\boldsymbol{s}}^{*}}^{\boldsymbol{g}}}{\displaystyle \sum_{\boldsymbol{s}}}{\boldsymbol{w}}_{{\boldsymbol{s}}^{*}}^{\boldsymbol{g}}\left(\boldsymbol{s}\right){\left({\boldsymbol{y}}_{\boldsymbol{s}}^{\boldsymbol{g}}-{\boldsymbol{X}}_{\boldsymbol{s}}^{\boldsymbol{g}}{\boldsymbol{\beta}}_{{\boldsymbol{s}}^{*}}^{\boldsymbol{g}}\right)}^{\boldsymbol{T}}\left({\boldsymbol{y}}_{\boldsymbol{s}}^{\boldsymbol{g}}-{\boldsymbol{X}}_{\boldsymbol{s}}^{\boldsymbol{g}}{\boldsymbol{\beta}}_{{\boldsymbol{s}}^{*}}^{\boldsymbol{g}}\right)+\boldsymbol{\lambda} \parallel {\boldsymbol{\beta}}_{{\boldsymbol{s}}^{*}}^{\boldsymbol{g}}{\parallel}_1 $$

Here, the weight ${w}_{s^{*}}^g(s)$ for samples in subtype ***s*** when we estimate the coefficients of gene ***g*** in subtype ***s*** ^*^ is defined as *K*_*h*_(*dist*(*s*, s*)) where *K*_*h*_ is a symmetric kernel function, *h* is the kernel bandwidth, and *dist*(*s*, s*) is a distance between samples from subtype *s* and s*. Note that the entire samples from all the subtypes are used for estimation of ${\boldsymbol{\beta}}_{{\boldsymbol{s}}^{*}}^{\boldsymbol{g}}$ including samples from subtype s* but with different contribution to the final estimation. For the proper control of subtype-specific estimation, samples are learned at different rate based on target estimates.

The challenging problem is to set the geographical distance between heterogeneous samples to be applied in forms of kernel. We assumed that given two observations have different distribution over DNA methylation genes in which expression level is affected by subtype-specific molecular process. From the fact that two samples are not originated from the same distribution, the distance between them can be measured by conducting Kolmogorov Smirnov (K-S) test. K-S test is used to decide if given two samples come from a population with a specific distribution. The advantage of K-S test is that the distribution of the K-S test statistic itself does not depend on the underlying cumulative distribution function being tested. Taking advantage of this fact, it is intuitive to set the critical value as distance between two samples. Finally, kernel weighting is applied to weighted lasso regression based on the distance. Radical Basis Function (RBF) kernel of *K*_*h*_(*d*) = exp(−*d* ^2^/*h*) is used to give different weights to each observation based on their distance [33]. That is, ${w}_{s^{*}}(s)$ is defined as exp(−*distance* ^2^/*h*) where *distance* is the critical value resulted from K-S test between samples from subtype *s* and s*, and *h* is the kernel bandwidth that is tuned through cross-validation.

## Results

### Prediction of gene expression level based on methylation profiles

**Fig. 2:**
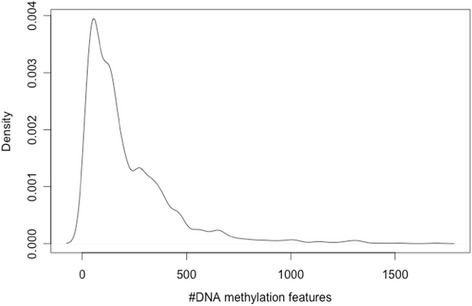
Density plot for the number of DNA methylation features across all target genes. The number of methylation features ranges from 10 to 1698. For most of target genes, around 200 ~ 300 number of features are used for estimation

As described in Methods, a covariate matrix ***X***_***s***_^***g***^ to estimate a target gene ***g*** is built by picking up methylation features from KEGG pathways that the target gene belongs to. The number of selected features ***p*** ^***g***^ varied across target genes, which is around 200 ~ 300 on average, 10 at minimum, and 1698 at maximum. Figure 2 shows the density plot for the number of features to predict target genes.

One of the advantages of our proposed method is that different sets of well-estimated genes having little prediction error can be obtained from subtype-specific estimation. It is observed that genes that might be sensitive to breast cancer subtype show prediction improvements when estimated using kernel weighted lasso. For validation of subtype-specific estimation over target genes, we pick up top 10 well-estimated genes over entire target genes as shown in the column *Overall* in Table 2, and then pick up top 10 better-estimated genes that have smaller prediction error in our proposed method than in the plain [34] lasso framework. We observe that most of the genes shown in four different subtypes do not appear in Overall. It means our proposed method is capable of recovering genes affected by breast cancer subtype that plain lasso cannot detect.

**Table 2:** Top 10 well-estimated gene list

Overall	HER2 positive	Luminal A	Luminal B	Triple negative
TRA2B	MMP1	PSMD3	PSMD3	ABCC12
HNRNPK	SPDYC	GPD1	CDC6	SLC18A2
RAB5B	ERBB2	ERBB2	RPL19	IVD
HNRNPK.1	ELOVL2	UGT1A6	ERBB2	ABCA12
HNRNPK.2	SERPINA5	BMPR1B	CACNG6	DNALI1
SEC11A	NPY1R	CTSE	PCK1	DEGS2
SF3A1	SEMA3E	CCL21	PSMB3	UCHL1
SRP14	AKR1B10	TAT	PIP4K2B	NEIL1
CDC42	UGT8	RPL19	CALML3	MAGOH
NRF1	EPO	ATP6V0A4	ABCC12	HGD

Genes having the smallest prediction error over all target genes are shown in column Overall, and genes that show prediction improvement when estimated using kernel weighted lasso over the plain lasso are shown for each subtype in the remaining columns

Furthermore, we examine pathway-based prediction performance over subtypes to identify the impact of our proposed method on pathway analysis. The performance on a pathway is measured by summing up error rates of target genes that belong to the pathway. In Table 3, 20 well-estimated KEGG pathways over entire subtypes are listed. And Table 4 represents top 10 pathways better estimated than the one without subtype information. We observe that commonly well-estimated pathways in Table 3 are not seen in Table 4. As discussed in [35], ERBB2 gene amplification and overexpression of the ERBB2 tyrosine kinase receptor is shown in breast cancer. [34] observed the upregulation of NPY1R is associated with ER^+^ breast cancer. Also, UCHL1 expression in invasive ductal carcinomas significantly correlated with the triple negative phenotype [36]. Previous researches show more than 5 genes at subtype columns are known to affect breast cancer subtype directly or indirectly. Especially genes in Triple negative are associated with breast cancer subtype progression.

**Table 3:** Top 20 well-estimated KEGG pathways

Overall	
SPLICEOSOME	SNARE_INTERACTIONS_IN_VESICULAR_TRANSPORT
PROTEIN_EXPORT	VALINE_LEUCINE_AND_ISOLEUCINE_BIOSYNTHESIS
AMINOACYL_TRNA_BIOSYNTHESIS	UBIQUITIN_MEDIATED_PROTEOLYSIS
NON_HOMOLOGOUS_END_JOINING	DNA_REPLICATION
RNA_DEGRADATION	REGULATION_OF_AUTOPHAGY
NUCLEOTIDE_EXCISION_REPAIR	RENAL_CELL_CARCINOMA
BASAL_TRANSCRIPTION_FACTORS	GLYOXYLATE_AND_DICARBOXYLATE_METABOLISM
MISMATCH_REPAIR	OXIDATIVE_PHOSPHORYLATION
RNA_POLYMERASE	NOTCH_SIGNALING_PATHWAY
PROTEASOME	PARKINSONS_DISEASE

**Table 4:** Top 10 better-estimated KEGG pathways per subtype

HER2 positive	Triple negative
GLYCOLYSIS_GLUCONEOGENESIS	GLYCOLYSIS_GLUCONEOGENESIS
CITRATE_CYCLE_TCA_CYCLE	CITRATE_CYCLE_TCA_CYCLE
PENTOSE_PHOSPHATE_PATHWAY	PENTOSE_PHOSPHATE_PATHWAY
FRUCTOSE_AND_MANNOSE_METABOLISM	PENTOSE_AND_GLUCURONATE_INTERCONVERSIONS
GALACTOSE_METABOLISM	FRUCTOSE_AND_MANNOSE_METABOLISM
FATTY_ACID_METABOLISM	GALACTOSE_METABOLISM
STEROID_BIOSYNTHESIS	ASCORBATE_AND_ALDARATE_METABOLISM
PRIMARY_BILE_ACID_BIOSYNTHESIS	FATTY_ACID_METABOLISM
OXIDATIVE_PHOSPHORYLATION	STEROID_BIOSYNTHESIS
PURINE_METABOLISM	PRIMARY_BILE_ACID_BIOSYNTHESIS
Luminal A	Luminal B
GLYCOLYSIS_GLUCONEOGENESIS	GLYCOLYSIS_GLUCONEOGENESIS
PENTOSE_PHOSPHATE_PATHWAY	CITRATE_CYCLE_TCA_CYCLE
PENTOSE_AND_GLUCURONATE_INTERCONVERSIONS	PENTOSE_PHOSPHATE_PATHWAY
FRUCTOSE_AND_MANNOSE_METABOLISM	PENTOSE_AND_GLUCURONATE_INTERCONVERSIONS
GALACTOSE_METABOLISM	FRUCTOSE_AND_MANNOSE_METABOLISM
ASCORBATE_AND_ALDARATE_METABOLISM	GALACTOSE_METABOLISM
FATTY_ACID_METABOLISM	ASCORBATE_AND_ALDARATE_METABOLISM
STEROID_BIOSYNTHESIS	FATTY_ACID_METABOLISM
PRIMARY_BILE_ACID_BIOSYNTHESIS	STEROID_BIOSYNTHESIS
STEROID_HORMONE_BIOSYNTHESIS	PRIMARY_BILE_ACID_BIOSYNTHESIS

### Subtype-specific prediction performance

**Fig. 3:**
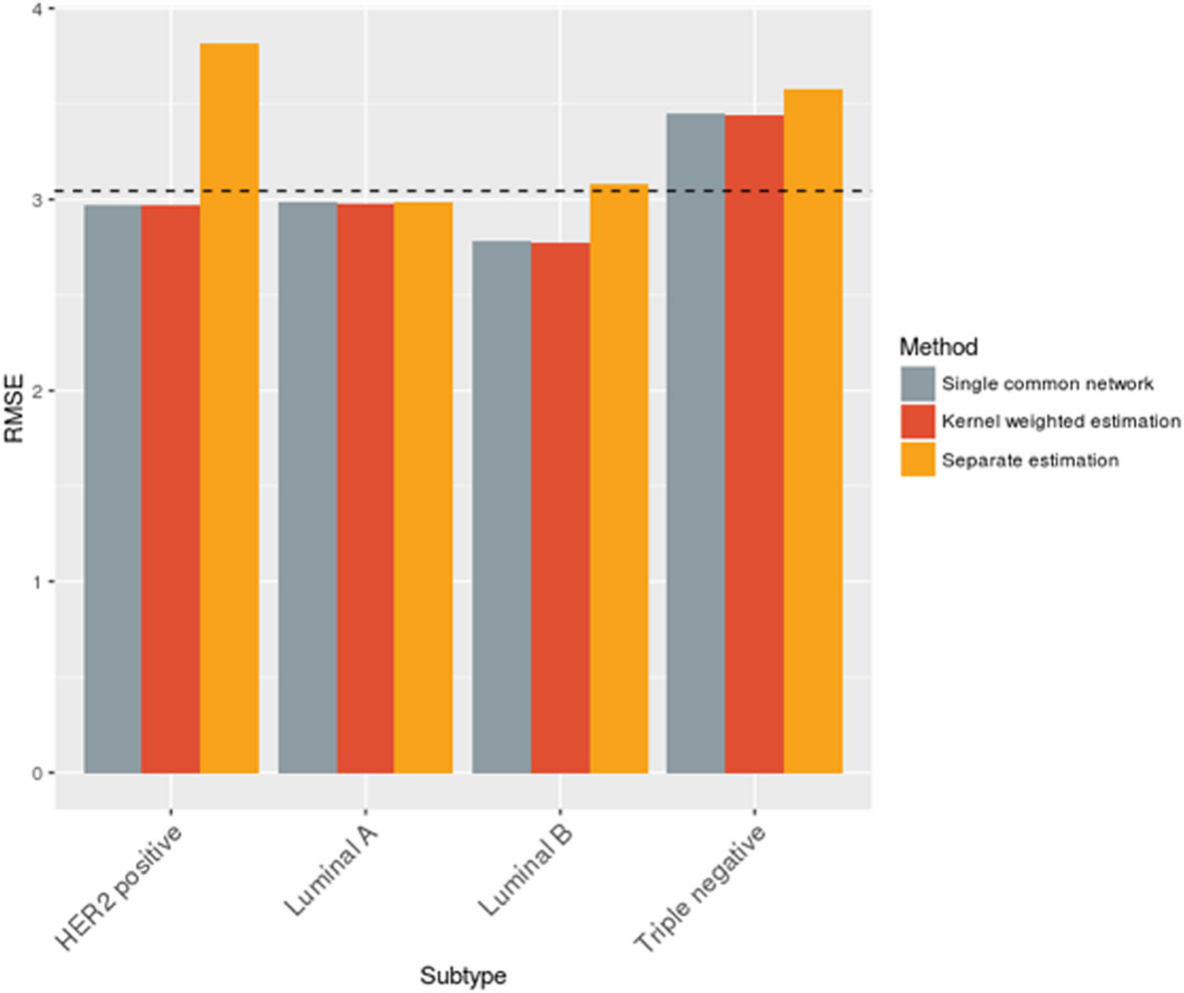
Subtype-specific Root Mean Squared Error from 5-fold cross validation. Each bar represents the average prediction error obtained from the proposed method (red), separate estimation that uses only the corresponding subtype data (yellow), and a single common estimation ignoring the subtype information (gray). Our proposed method shows significantly improved performance over the separate estimation approach, and slightly better or comparable performance over single common estimation

Next, we compare the subtype-specific prediction performance of the proposed method with two baseline approaches: one in which each subtype data are learned separately with plain lasso framework, and the other for entire data learned equally without weighting using plain lasso. Figure 3 represents Root Mean Squared Error (RMSE) from 5-fold cross validation, resulted from each approach over entire target genes. Note that dotted horizontal line is the mean of error rates over entire genes estimated by plain lasso without kernel weighting. As seen in Fig. 3, our proposed method shows substantial prediction improvement over separate estimation approach. Especially in case of HER2 positive subtype that has the smallest number of samples of 16, our kernel-weighted approach outperforms separate estimation the most significantly. This result is as expected because our proposed method can effectively enlarge the sample size by incorporating information in samples from different subtypes. On the other hand, the largest subtype Luminal A with 307 samples does not show much performance improvement because the number of samples is already large enough for estimation. We find that the overall accuracy of our proposed method is comparable to the one for estimating a single common network (gray bars in Fig. 3) that does not produce subtype-specific association networks.

### Subtype-specific association network

**Fig. 4:**
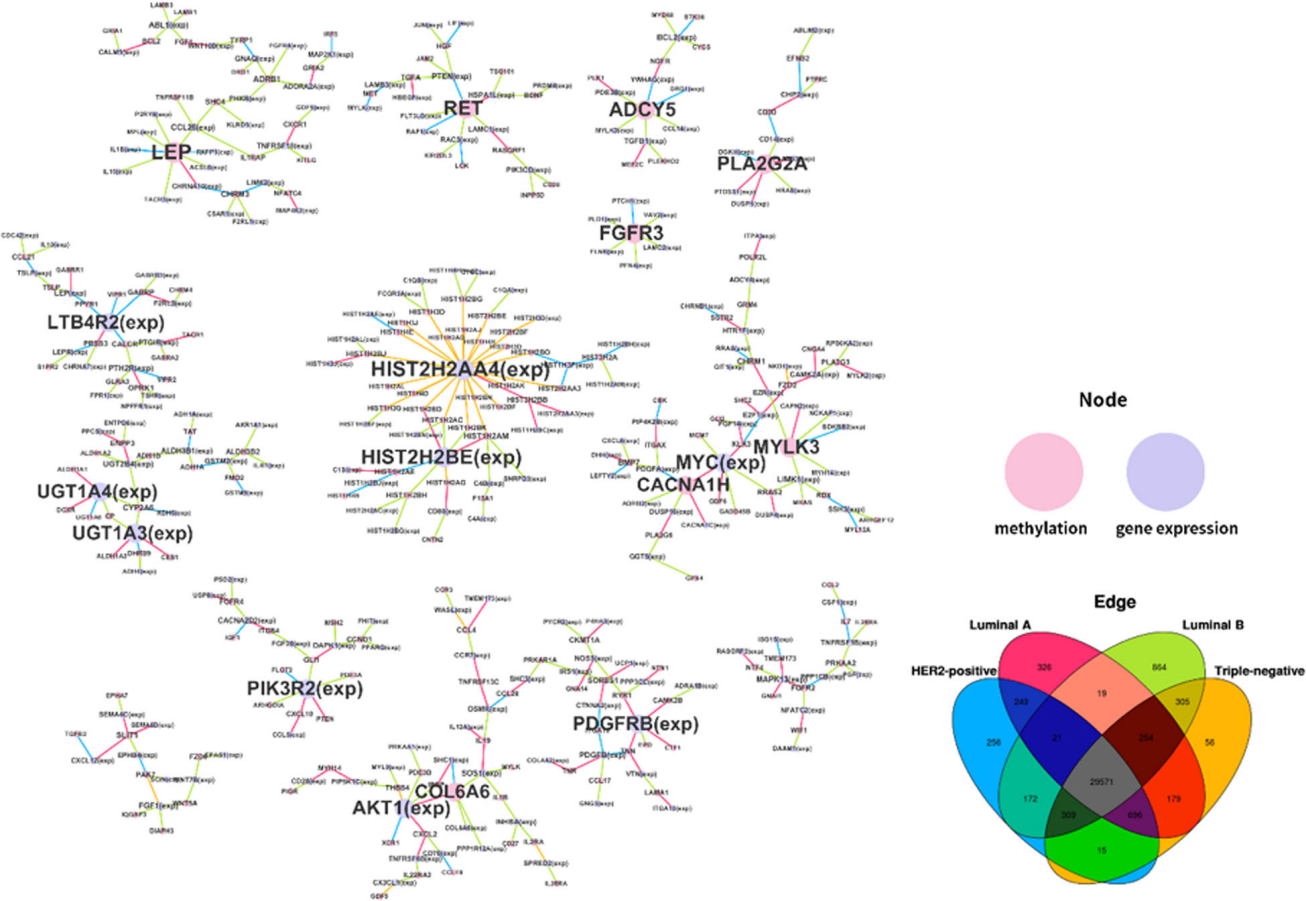
Subtype-specific association networks between DNA methylation and gene expression, and Venn diagram for the number of edges in the network. An edge in a subtype-specific association network is drawn if methylation node A and gene expression node B have non-zero a regression coefficient resulted from kernel weighted lasso. The edges are colored based on their subtype-specific association. Venn diagram represents the number of edges occurring in each association network where intersection region stands for the number of edges appearing in more than two networks

We construct subtype-specific association networks by using the regression coefficients estimated by the proposed method. The node represents either methylation feature or gene expression feature and the edge represents the subtype-specific association. That is, if node A is associated with node B under specific subtype having non-zero coefficients, the edge is drawn. Figure 4 illustrates the resulting association network between DNA methylation and gene expression genes. The total number of edges in each subtype network is 31289, 31306, 31515, and 31385 for HER2 positive, Luminal A, Luminal B, and Triple negative, respectively, among which 29571 number of edges (88.82%) are common across all the subtypes as shown in the Venn diagram of Fig. 4 (gray region). To look into only subtype-specific edges in the network, common edges in at least two or more subtypes are not shown. The hub genes, which have a large number of associated genes are represented as bigger-sized nodes. The four types of subtype-specific edges are marked with the color of each region in the Venn diagram of Fig. 4. Among 4,061 genes, 2,063 subtype-specific features and 1,502 number of association are observed. The numbers of subtype-specific edges are 256, 326, 864, and 56 for HER2 positive, Luminal A, Luminal B, and Triple negative, respectively.

We found that several hub methylation features in our subtype-specific association network are known to be involved in breast cancer progression. For example, LEP, the top hub methylation feature affecting the largest number of gene expressions with total degree of 9, is found to be associated with basal-like or luminal A breast cancer subtypes. Another example includes FGFR3 and FGFR4 that are known to be associated with breast cancer as revealed in [37]. Table 5 summarizes the top 5 hub methylation features and their subtype-specific degrees along with the supporting literature for the relevance of each feature in breast cancer.

**Table 5:** Top 5 hub methylation features in subtype-specific association network and their degrees

Gene Name	Total	HER2 positive	Luminal A	Luminal B	Triple negative	Literature
LEP	9	2	1	6	0	[63, 64]
RET	6	2	1	3	0	[65, 66]
FGFR3	6	1	0	5	0	[37, 67]
PLA2G2A	6	1	3	2	0	
ADCY5	6	2	0	4	0	

For each methylation node, the total number of connected edges that are present over four subtype-specific association networks is shown in the column Total. Remaining columns represent the degrees in the corresponding subtype-specific association network

## Discussion

The proposed kernel weighted model allows subtype-specific prediction of gene expressions based on methylation data along with discovery of subtype-specific association patterns between them even when the number of samples per subtype is substantially small. The reduction in error across the subtype given by the model was the starkest in genes coding for GTPases, transcription factors, and splicing factors, and nucleic acid binding proteins. Given our model’s incorporation of factors at the transcriptome-epigenome level, incorporating such epigenetic signals into the model improved subtype prediction and recapitulates the importance of RNA processing mechanisms, transcription factors, and metabolic processes in determining subtype beyond the genomic level.

The RMSE over all subtypes using the proposed prediction model was lowest for genes coding for transcription factors, GTPases, and nucleic-acid binding proteins: TRA2B, HNRNPK, RAB5B, SEC11A, SF3A1, SRP14, CDC42, and NRF showed the lowest RMSE over all breast cancer subtypes. This is consistent with the fact that our kernel-weighted model incorporates epigenomic information and proof of concept of the potential of the incorporating previously-overlooked epigenomic information in cancer subtype classification. HnRNP K showed the second lowest prediction error over all subtypes in the kernel-weighted model; HnRNP K is a multifunctional protein that binds the TATA-box [38] and is associated with both oncogenic and tumor-suppressor pathways [39] by interacting with many kinases including ncRNAs to control the expression of target genes [40]. TRA2B, SF3A1, and NRF1 were splicing factors that showed significant improvement in subtype prediction when epigenomic data were incorporated. TRA2B showed the lowest prediction error over all subtypes and had previously been shown to be specifically induced in breast cancer, and more induced in invasive breast cancers compared to non-invasive breast cancers, perhaps by splicing CD44 isoforms [41]. When both TRA2A and TRA2B are eliminated, expression of full-length CHK1 protein is reduced [42]. Polymorphisms in SF3A1 have been found to be associated with slightly higher colorectal cancer risk [43] and breast cancer [44]. Lastly, NRF1, a splicing factor was shown to be part of a redox signaling pathway where PTEN and CDC25A were modified by reactive oxygen species, leading to activation of NRF1 and estrogen-induced growth of breast cancer cells [45] and NRF1 was previously included in a Bayesian model of transcription factors involved in estrogen receptor alpha (ER-a). In breast cancer cells with acquired resistance to tamoxifen, the ER-a network (of which NRF1 is a component) lost responsiveness to 17-beta-estradiol; this loss of responsiveness was mediated by epigenomic changes [46]. This indicates the fundamental importance of epigenomics in modifying the transcription and translation of multi-functional proteins and genes involved in the induction of an oncogenic phenotype.

The weighted estimation model also showed marked improvement in marking the influence of GTPases in accurately predicting breast cancer subtype. Two small GTPases, CDC42 (Rho) and RAB5B (Ras) were among the ten genes with smallest RMSE across all subtypes. CDC42 is a locally excitable GTPase which steers cells during chemotaxis [47] and induces the extension of filopodia [48]. In the developing mammary gland, overexpression of CDC42 induces hyperbranching, increased stromal thickness and collagen deposition, and elevated mRNA expression of extracellular matrix proteins in stromal cells [49]. MiR-1 binding with CDC42 (mediated by MALAT1) induced migration and invasion of breast cancer cells [50] and CDC42 activity has been implicated in the invasive phenotype [51]. CDC42 is overexpressed in a variety of tumor types and is activated by oncogenic Ras protein to instigate Ras-mediated tumorigenesis in colon cancer [52]. Another GTPase that showed improvement in predictivity after incorporating epigenetic modification was RAB5B, a Ras GTPase that participates in the early stages of endocytosis. The early endosomal autoantigen EEA1 was found in a yeast two-hybrid system to interact directly with RAB5B in a GTP-dependent matter, independent of intrinsic GTPase activity [53]; in tumor cells, exosomes tended to localize with EEA1 [54]. Suppression of *RAB5A* and *RAB5B* hampered the degradation of *EGFR*, an epidermal growth factor receptor [55]. *RAB5B* specifically interacts with *LRRK2* (mutations in which are associated with autosomal-dominant Parkinson’s disease) and can rescue synaptic vesicle endocytosis defect induced by *LRRK2* knockout [56]. Administration of paclitaxel at 60 ng/mL in breast cancer cells caused significant increase in the expression of the *RAB* family of genes in comparison to the control group. *RAB5B* with lost GTPase function in lymphocytes caused the formation of abnormal, giant hybrid organelles which showed changed morphology over time [57]. The influence of epigenomic data recapitulates the importance of incorporating multi-omics data when constructing complex disease models, subtypes, and classifications.

The network illustration (Fig. 4) implicated multiple levels and mechanisms by which epigenetic features impact subtype classification, especially on the histone, nucleosome, and cellular differentiation levels. HIST2H2AA4 is a variant of histone 2A (specifically, type 2-A) that is implicated in histone core octamer stabilization; Histone 2A forms a dimer with Histone 2B, and then forms a tetramer with the H3-H4 dimer [58]. It was found that HIST2H2AA4’s interaction with various linker histones, especially variants of H1. Among core histones, histone H2A has by far the maximum number of variants (totaling 19). The exact role of HIST2H2AA4 in the breast cancer phenotype merits additional investigation given that it was previously implicated in a study of genes that statistically distinguish the hyperthermic response of three breast cancer lines compared to normal mammary epithelial cells [59]. The interaction between an element of Collagen VI (COL6A6) and serine-threonine protein kinase AKT1 was also found to be meaningful in a search for significant networks that included epigenetic data. AKT1 encodes a serine-threonine protein kinase which is activated by platelet-derived growth factor which has been implicated in many cancers, with the highest incidence in breast cancer [60]. A subset of breast cancer specimens was found to only contain AKT1 as a driver alteration, although AKT1-mutants were also often found to contain mutations in other driver genes [61]. Down-regulation of the Collagen VI extracellular matrix by AKT1 and upregulation of MMP1 was found in human dermal fibrolasts [62]; our model incorporating epigenetic control also reduced error in MMP1 the most when predicting a HER2 positive subtype (Table 2).

In terms of the model accuracy for predicting the gene expression level, our proposed methodology shows performance improvement only to part of target genes, that is, the kernel weighted method does enhance the prediction accuracy for entire target genes. As shown in Fig. 3, large improvement over single common estimation in terms of prediction accuracy is not observed. That is because genes that are not sensitive to breast cancer subtype may not benefit much from the proposed method.

## Conclusions

In this study, we proposed a novel method for identifying subtype-specific gene expressions based on DNA methylation profiles. To make it possible to provide subtype-specific association network, kernel weighted lasso model is applied in which breast cancer subtype information is employed in forms of kernel. We found our proposed method is able to discover subtype-sensitive genes that plain lasso framework could not detect (Table 2). Especially for the subtype with small sample size, it outperforms the separate estimation method substantially. Furthermore, our framework provides a subtype-specific network, which represents genomic association underlying breast cancer subtypes. From the perspective of observations, we assumed samples from different subtypes come from different distribution. The distance between samples from different subtypes are set based only on their distribution. Thus, for our future work, well-designed kernel that appropriately reflects association exerted between samples will enhance the performance, and can reveal the relationship between samples.
